# Molecular Interactions Driving Intermediate Filament Assembly

**DOI:** 10.3390/cells10092457

**Published:** 2021-09-17

**Authors:** Pieter-Jan Vermeire, Giel Stalmans, Anastasia V. Lilina, Jan Fiala, Petr Novak, Harald Herrmann, Sergei V. Strelkov

**Affiliations:** 1 Laboratory for Biocrystallography, KU Leuven, 3000 Leuven, Belgium; pieterjan.vermeire@kuleuven.be (P.-J.V.); giel.stalmans@kuleuven.be (G.S.); anastasia.lilina@kuleuven.be (A.V.L.); 2 Department of Biochemistry, Charles University, 12800 Prague, Czech Republic; jan.fiala@biomed.cas.cz (J.F.); pnovak@biomed.cas.cz (P.N.); 3 Institute of Microbiology of the Czech Academy of Sciences, 14220 Prague, Czech Republic; 4 Institute of Neuropathology, University Hospital Erlangen, Friedrich-Alexander University Erlangen-Nürnberg, 91054 Erlangen, Germany; Harald.Herrmann-Lerdon@uk-erlangen.de

**Keywords:** X-ray crystallography, assembly, chemical analytical cross-linking, intermediate filament, keratin, vimentin, lamin, cryoelectron microscopy

## Abstract

Given the role of intermediate filaments (IFs) in normal cell physiology and scores of IF-linked diseases, the importance of understanding their molecular structure is beyond doubt. Research into the IF structure was initiated more than 30 years ago, and some important advances have been made. Using crystallography and other methods, the central coiled-coil domain of the elementary dimer and also the structural basis of the soluble tetramer formation have been studied to atomic precision. However, the molecular interactions driving later stages of the filament assembly are still not fully understood. For cytoplasmic IFs, much of the currently available insight is due to chemical cross-linking experiments that date back to the 1990s. This technique has since been radically improved, and several groups have utilized it recently to obtain data on lamin filament assembly. Here, we will summarize these findings and reflect on the remaining open questions and challenges of IF structure. We argue that, in addition to X-ray crystallography, chemical cross-linking and cryoelectron microscopy are the techniques that should enable major new advances in the field in the near future.

## 1. Structural Principles and Biological Role of IFs

Intermediate filaments (IFs) together with actin microfilaments (MFs) and microtubules (MTs) are the three main cytoskeletal filament systems found in metazoan animals. In contrast to the much more rigid and fragile MTs and MFs, IFs have unique properties such as lack of polarity, extreme resilience, and extensibility [1,2]. 

Humans carry over 70 different IF proteins that are classified into five major types according to their amino-acid sequence. For an extensive reference on human IF protein sequences including disease variants, see Szeverenyi et al. [3] and the website address provided in this paper. Of the five IF homology types, various keratins make the first (acidic keratins) and the second ones (neutral/basic keratins). The third homology type includes vimentin, desmin, glial fibrillary acidic protein (GFAP), and others. Neurofilaments constitute the fourth IF type [4]. While most IF proteins form homodimers in solution, a characteristic feature of keratins is the highly preferential heterodimerization of type I and type II chains. Consequently, mature keratin IFs always contain both chain types. For cytoplasmic IF proteins, further self-assembly of such homo- or heterodimers typically leads to smooth filaments with an average diameter of 10–12 nm. In contrast, the type V nuclear IF proteins (lamins) form much thinner filaments that are the main constituents of a fibrillar network (lamina) located at the inner nuclear membrane [5,6].

A signature feature of IF proteins is their central α-helical ‘rod’ domain. Structural organization of this domain is conserved across all IF types. However, the amino-acid sequence conservation of the rod domain is rather low, except for 20 residues at either end. This domain features a characteristic pattern of hydrophobic residues, which is responsible for the formation of an α−helical coiled-coil. The presence of a coiled-coil defines the elementary IF dimer, which has a diameter of 2–3 nm and a length of 45 nm for cytoplasmic and 52 nm for nuclear IF proteins. The rod domain is flanked by the non-α-helical N-terminal ‘head’ and C-terminal ‘tail’ domains. Both terminal domains include extensive regions of intrinsic disorder [7,8,9]. Importantly, the head domain plays an essential role in IF assembly. 

IF assembly is based on specific associations of the elementary dimers in two directions: the lateral (side-by-side) and longitudinal (head-to-tail). For cytoplasmic IFs, the first assembly intermediate is a tetramer that stays soluble in low ionic strength buffers at neutral pH such as 2 mM sodium phosphate buffer, pH 7.5 [10]. Upon increase in the ionic strength in vitro, the tetramers very rapidly associate laterally yielding so-called ‘unit-length filaments’ (ULFs) [10,11,12,13,14]. ULF formation happens within one second, and is followed by a much slower elongation step by longitudinal association of multiple ULFs [13,15,16]. For human vimentin, the assembled filaments were reported to contain, on average, 32 monomers in the cross-section [12]. Whereas the filaments are initially rather loosely packed, the following radial compaction step results in mature filaments with a diameter of 10–12 nm within the first 15 min of assembly. Of note, as of today, the exact 3D architecture of mature IFs is still a subject of debate. As we will discuss here, different possibilities exist with respect to the arrangement of the tetramers. 

Assembly of nuclear lamins is very distinct from that of cytoplasmic IFs. As demonstrated in vitro, lamin dimers have the capacity to associate longitudinally to form longer head-to-tail threads, which can further associate [6,17,18,19]. In particular, 3.5 nm wide lamin filaments containing two antiparallel dimeric threads have been observed both in vitro and ex vivo [5,6,20]. In addition, lamins readily form paracrystals in vitro [18]. Just like the thicker cytoplasmic IFs, the structure of lamin filaments is not yet fully understood in atomic detail.

Finally, it should be stressed that IFs are highly dynamic structures. In living cells, the IF network needs to undergo major rearrangements to enable specific processes such as mitosis, migration, and apoptosis [21]. Moreover, individual cytoplasmic IFs were shown to be highly extensible. This feature explains the important contribution of the IF network to cellular plasticity, which provides protection from external mechanical stresses. IF dynamics is regulated via multiple signaling cascades through various post-translational modifications (PTMs), which are cell cycle or developmental phase specific [22]. For instance, during mitosis, site-specific phosphorylation plays a key role in IF disassembly [23,24,25]. Of note, it is the dynamic character of IFs that further complicates their structural studies.

In this review, we will discuss the current knowledge on the structure of cytoplasmic and nuclear IFs, especially focusing on the data available on the interdimer interactions driving the filament assembly. We will start by summarizing the atomic resolution data on the dimers and tetramers, which could be obtained using X-ray crystallography and related techniques. Thereafter, we will discuss the utility of analytical chemical cross-linking toward unravelling the dimer–dimer interactions. The main bulk of cross-linking data were obtained back in the 1990s and early 2000s on several cytoplasmic IFs. However, major questions remain with respect to the use of these data toward producing a full 3D model of the 10 nm filament. For nuclear IFs, such cross-linking experiments have only been reported recently. These data were obtained using modern mass-spectrometry based identification of cross-linked peptides and are more detailed. However, the consensus on the underlying molecular features such as the correct alignment of the dimers within the 3.5 nm nuclear filament is still lacking. 

## 2. Atomic Structure of the Elementary IF Dimer and A_11_ Tetramer 

X-ray crystallography is a leading technique toward unravelling biological structure at atomic resolution. The main limitation of this technique is the necessity of obtaining suitable crystals. With regard to IF components, successful crystallization of full-length proteins is very unlikely due to their elongated shape and flexibility as well as the presence of intrinsically disordered head and tail domains [26]. To circumvent these problems, a ‘divide-and-conquer’ crystallographic approach based on using shorter fragments of the IF rod domain was proposed a while ago [27]. This approach enabled crystallization and X-ray structure determination for larger parts of the dimer for representatives of major IF classes including vimentin, lamin A, and several keratins [7,8]. The obtained crystallographic data could be cross-checked and complemented by other experimental techniques, as discussed below.

### 2.1. Elementary Dimer 

Analysis of the primary sequence of the IF rod domain reveals a pronounced heptad repeat pattern, highly suggestive of α-helical coiled-coil, a key protein structure motif. Coiled-coil formation is the driving force of homo- or heterodimerization of IF chains as well as the reason for the elongated shape of the elementary dimer. Further detail on the principles of the coiled-coils can be found in Box 1 and Figure 1 of Chernyatina et al. [7]. 

Early sequence analyses have pointed to three extended α-helical regions in the IF rod domain. By now, using X-ray crystallography and other techniques, these three regions (i.e., coil1A, coil1B, and coil2) could be convincingly confirmed [7]. The coiled-coil regions are interconnected by short and presumably flexible, mostly non α-helical linkers L1 and L12. Importantly, the lengths of the three coiled-coil segments are largely conserved across all IF classes. One exception is a 42-residue longer coil1B segment in lamins and cytoplasmic IF proteins of invertebrates [28] compared to cytoplasmic IF proteins of chordates and vertebrates [7]. 

Interestingly, besides the heptad-based regions that yield the canonical left-handed coiled-coil, the N-terminal portion of coil2 features an 11-residue (hendecad) pattern which results in a parallel, rather than twisted, α-helical bundle geometry. Here, it should be noted that an early application of a computer algorithm recognizing the heptad pattern has only predicted the N-terminal portion of coil2 to feature a short left-handed segment (coil2A) followed by an additional linker (L2). This possibility was analyzed theoretically in [29]. While experimental studies have since led to a convincing rejection of this model, the erroneous splitting of coil2 into coil2A and coil2B still propagates in the scientific literature to-date.

A systematic overview of all crystal structures obtained for IF dimer fragments can be found in [7] and in updated form in [8]. Technical details, pearls, and possible pitfalls of crystallographic studies were discussed in [4,26]. One common challenge of shorter rod domain fragments is that they may not yield parallel, in-register dimeric coiled-coils [26,30]. Therefore, it is advisable to check for the correct fragment oligomerization by means of biophysical techniques such as gel filtration coupled to multi-angle light scattering. Although many of the produced rod fragments were found to form the correct parallel coiled-coil dimers, aberrant structures such as trimers and antiparallel arrangements were observed [7,31,32]. 

The solution to this problem could be found in fusing the short fragments with either an N- or C-terminal capping motif that would ‘bootstrap’ the formation of a correct dimeric, parallel, and in-register coiled-coil. This approach had been originally developed for coiled-coil fragments of myosin [33] and more recently applied to nuclear lamins [30]. A particularly efficient capping motif may include a cysteine residue that forms a disulfide bridge across the two chains. Toward crystallographic phasing, the capping motif alone is often sufficient to provide the initial phase estimate through molecular replacement. Alternatively, a disulfide bridge located on the dimer axis is capable of producing a relatively strong anomalous signal that can also be used for phasing [30].

**Figure 1:**
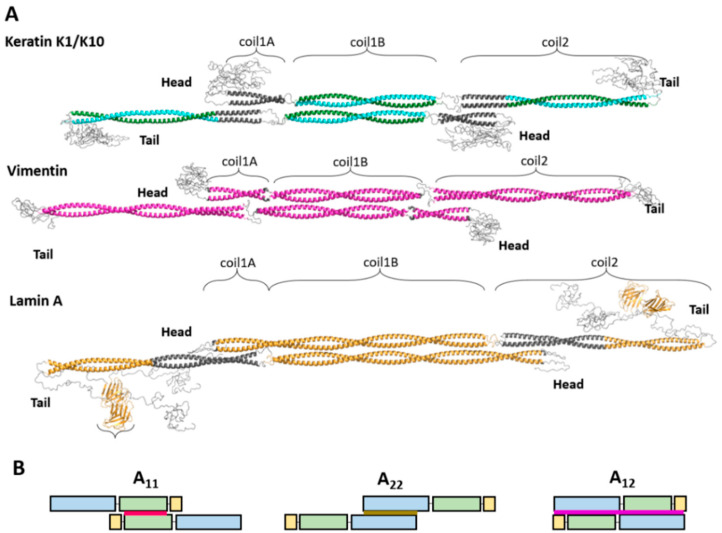
(**A**) Atomic models of the tetramer for representatives of several IF types. For individual dimers, regions with established crystal structures are shown in color. The coiled-coil regions that are a result of in silico modeling using the program CCFold [34] are shown in dark gray. The antiparallel arrangement of two coil1B domains has been resolved crystallographically in each of the three cases. (**B**) Schematic illustration of different lateral dimer alignment modes seen in mature cytoplasmic filaments. Coil1A is shown in yellow, coil1B in green, and coil2 in blue. The A_11_ mode (red) is defined by an antiparallel overlap of coil1B regions. A_22_ is defined by the antiparallel alignment of coil2. A_12_ (purple) corresponds to an antiparallel unstaggered alignment.

Taken together, crystal structures of individual fragments enable the reconstruction of the nearly complete atomic structure of the vimentin rod domain. There, all three α-helical segments have been resolved, while the only region resisting crystallization to date is the linker L12. The model of the vimentin dimer is shown in Figure 1B (as part of the soluble A_11_ tetramer to be discussed below). Somewhat less complete coverage by crystal structures of individual fragments has also been achieved for the rod domains of keratins and lamins (Figure 1). 

Of note, coiled-coil structures can be efficiently modeled in silico. A recently designed algorithm CCFold is capable of building such models by threading the accumulated crystallographic data on various coiled-coils [34]. This way, it becomes possible to model the atomic structure of the rod domain for any IF protein given its amino acid-sequence only. The resulting models show good agreement with the experimentally determined IF fragment structures [34].

Finally, the head and tail domains typically contain large regions of intrinsic disorder and vary highly in both length and sequence among different IF proteins [7,20]. In particular, the head domains often contain positively charged regions, as opposed to the mainly acidic nature of the rod domains. At the same time, the terminal domains often carry specific functional regions such as the characteristic IgG fold within the tail domain of lamins [35] or phosphorylation sites in the vimentin head [36,37]. Moreover, some evidence suggests that the disordered terminal regions play a key role in filament assembly, and that these regions become more structured in mature filaments [8,9,38,39,40].

While crystallography remains the main source of atomic detail of the IF rod structure, an important complementary approach is the use of electron paramagnetic resonance on site-directed spin-labelled samples (SDSL-EPR) [26]. This technique has been used before on both coiled-coil domains as well as on flexible head and tail domains of full-length vimentin [41,42,43,44,45,46,47,48]. 

### 2.2. A_11_ Tetramer of Cytoplasmic and Nuclear IF Proteins

A tetramer is a typical soluble form of cytoplasmic IF proteins. In particular, recombinant human vimentin forms stable tetramers in low molarity neutral buffers [10]. SDSL-EPR studies [7,26,49] revealed that such tetramers contain two dimers aligned in antiparallel and half-staggered fashion with coil1B domains approximately in register. Here, the residue 191 of all chains in the middle of coil1B are located at the symmetry dyad of the tetramer (Figure 1A). Importantly, this corresponds to the so-called A_11_ alignment that was established through chemical cross-linking by Peter Steinert and colleagues in mature filaments [7,10,50]. In addition, these authors discovered two other modes of lateral dimer alignment. The A_22_ mode, which corresponds to a similar half-staggered association of an antiparallel pair of dimers but with coil2 domains aligned. The A_12_ mode corresponds to an unstaggered dimer association (Figure 1B) (see next section). Additionally, the length of vimentin ULFs, as visualized using negatively stained electron microscopy (EM) (~66 nm), matches the expected length of the A_11_ tetramer [10]. 

Notably, the A_11_ type tetramer can be formed by a vimentin fragment corresponding to coil1B alone, as evidenced by its crystal structure [49], followed by a very similar tetrameric structure for the coil1B fragment of GFAP. Along with keratin heterodimers, the A_11_ alignment was clearly seen in cross-linking studies, while the crystal structure of a K1/K10 fragment corresponding to coil1B revealed the same arrangement [51,52,53,54]. 

The antiparallel contact between the coil1B regions seen in A_11_ tetramers of cytoplasmic IF proteins is stabilized by hydrogen-bonding, salt bridges, electrostatic and hydrophobic interactions. Specifically, the structure reveals a conserved hydrophobic ‘knob’ near the C-terminus of coil1B, which is inserted in a hydrophobic pocket located at the opposite end of the tetrameric overlap. A double mutation of two hydrophobic residues within this anchoring knob to alanine resulted in the loss of tetramerization of coil1B segments, and also had a detrimental effect on the assembly of the full-length proteins, as shown for both keratins and vimentin [51]. Moreover, as demonstrated through site-directed mutagenesis, a small hydrophobic stripe on the surface of specific type I keratins contributes to tetramer stability, even though these hydrophobic stripe mutants assemble identically to the WT in vitro [55].

In contrast to cytoplasmic IF proteins, lamins were originally observed to form dimers in solution, although this required the presence of 300 mM NaCl. Upon reduction of ionic strength to ~150 mM, such dimers made long dimeric threads in a head-to-tail fashion [18,19]. Thus, under these conditions, the longitudinal assembly of lamins dominated over the lateral assembly. However, A_11_ type interaction has recently also been found in lamins. Of note, the lamin coil1B dimer contains 42 extra residues with respect to cytoplasmic IF proteins and thus an isolated coil1B dimer corresponds to 1.5 turns of the superhelix, compared to only one turn in cytoplasmic IF proteins (Figure 1). Nevertheless, crystallographic studies have consistently revealed A_11_ tetramers for both the isolated coil1B construct of lamin A [54] and a longer construct including the first 300 residues of the same protein [53]. Moreover, the interactions stabilizing the A_11_ tetramer in lamins are mostly similar to those in cytoplasmic IFs. In particular, the hydrophobic ‘knob-into-hole’ interaction is preserved. 

## 3. Use of Chemical Cross-Linking to Reveal the 3D Architecture of Complete Filaments

Beyond the tetramer level, structural studies of IFs are still presenting a major challenge. As we discuss below, it is the increasing level of disorder at higher assembly levels that highly complicates the structural studies. Under these circumstances, the analytical chemical cross-linking technique presents one of the few efficient options. Back in the 1990s, Steinert and colleagues put much effort into analyzing several cytoplasmic IF proteins using cross-linking. As a result, a bulk of data on the arrangement of dimers in cytoplasmic IFs could be obtained. In fact, these data have been widely regarded as establishing the basis of IF architecture ever since [8,56]. Interestingly, it is only in the last few years that new chemical cross-linking results became available, this time, on nuclear IFs [30,53,57]. In this section, we will cover both the technical aspects of the technique and its impact on understanding the IF structure.

### 3.1. Starting Material for Cross-Linking Studies

Many important results on the mature filament structure were obtained in the past on in vitro assembled filaments. Indeed, recombinant IF proteins can be effectively obtained using standard expression hosts such as *E. coli* [58,59]. Typically, the expressed IF proteins are conveniently obtained in the form of inclusion bodies. After denaturation in 8 M urea, the proteins are additionally purified via ion exchange and stored at −80 °C. Prior to further studies, a thawed aliquot is dialyzed in a step-wise fashion into gradually lower urea concentrations, and finally into a low molarity neutral buffer [10].

Under such conditions, cytoplasmic IF proteins are typically present as soluble tetramers, as has been established using analytical ultracentrifugation and other methods [10]. Further assembly can be induced in a test tube by increasing the ionic strength or lowering the pH, resulting in octamers and later mature filaments. The progress of assembly can be readily monitored using EM with negative staining [7,11,58]. Interestingly, several point mutants of vimentin were shown to form normal-looking ULFs, but were totally or partially incompetent of longitudinal assembly [10,13,60]. This provided a convenient means toward the structural studies of the ULFs, including chemical cross-linking [10] but also other methods such as small angle X-ray scattering (SAXS) [60].

Of note, early EM studies suggested that in vitro assembled IFs are comparable to those purified from cells, although the homogeneity and exact preservation of the microscopic structure in the in vitro assembled filaments remained a subject of debate [58]. At the same time, the filaments isolated from living cells are obviously presenting a very attractive study object. However, heterogeneity was also observed for such filaments [61]. Several post-translation modifications are known to affect the assembly process of IF proteins [22]. In particular, phosphorylation induces filament disassembly and appears to act as an important regulatory mechanism [56]. 

### 3.2. Chemical Cross-Linking Approach

First, we will discuss the cross-linking procedure itself, since one should be aware of the methodological differences between the procedures used in the 1990s and today, in order to fully appreciate the scope and limitations of various datasets reported. Indeed, the current ubiquitous use of mass-spectrometry (MS) for cross-link identification has multiple benefits over the previously used approach. Readers mainly interested in the impact of these studies for the understanding of IF architecture may proceed immediately to the next section.

A chemical cross-linker (e.g., formaldehyde) covalently links two nearby moieties within a single protein structure or a protein complex. The cross-linker itself consists of two chemical groups with specific reactivity, separated from each other by a spacer group (‘chemical/molecular ruler’). The range of cross-linking (i.e., the maximal possible Cα–Cα distance between the two residues) depends on the length of this spacer as well as the lengths of the side chains involved. It should be noted that, for flexible regions, the cross-linking procedure may ‘freeze-in’ the proximity of certain residues, even if these residues are only occasionally found close enough to each other. This means that cross-linking should not always be interpreted as revealing a static picture of the studied biological system. 

Historically, homobifunctional cross-linkers based on an amino-reactive *N*-hydroxysuccinimide ester (NHS-ester) such as bissulfosuccinimidyl suberate, disuccinimidyl glutarate, or disulfosuccinimidyl tartrate [62] have mainly been used. They form covalent linkages between N-termini, lysine, serine, threonine, and tyrosine residues ranked from high to low reactivity, respectively [63]. The NHS-ester is a good leaving group after nucleophilic attack by an amino group. Of note, here, Tris-HCl buffer cannot be used since it contains primary amines that readily quench the activity of the cross-linker. 

In addition, it is possible to use heterobifunctional ‘zero-length’ cross-linkers based on carbodiimide moieties such as 1-ethyl-3-(3-dimethylaminopropyl)carbodiimide (EDC). This reagent will typically link N-termini and lysines to C-termini and acidic side chains. EDC links the side chains of two residues directly, hence the name ‘zero-length cross-linker’. With a maximum Cα-Cα distance of 15 Å, EDC cross-links produce the most efficient restraints for the downstream 3D modeling. However, the MS data obtained after such cross-linking are relatively difficult to interpret since no ‘signature’ exogenous moieties are present in the resulting set of peptides.

In order to increase the robustness of cross-link assignment, two main strategies have been introduced. Equimolar mixtures of isotopically labelled (e.g., deuterated) and non-labelled cross-linkers are often employed. In this case, the cross-linked peptides can be detected as characteristic doublets in MS and subsequent fragment spectra, facilitating automatic data processing. Recently, MS-cleavable NHS-ester based cross-linkers such as disuccinimidyl sulfoxide and disuccinimidyl dibutyric urea are often used [62,63,64,65]. Here, further gas-phase fragmentation of the cross-linked peptide takes place during the second-dimension MS run, generating a specific fingerprint in the MS/MS spectra. This facilitates the cross-link detection by dedicated software and helps to avoid false positives [62]. 

The chosen cross-linker is added in a molar excess to the protein solution. In principle, the molar excess of cross-linker over the protein should not exceed the number of reactive side chains [66]. Subsequently, reaction time, temperature, cross-linker excess, buffer, etc. can be optimized further by assessing the results using sodium dodecyl sulphate polyacrylamide gel electrophoresis (SDS-PAGE). Under optimal cross-linking conditions, such gels should show distinct cross-linked bands (dimeric or higher) but without too much over-cross-linking, resulting in high molecular weight species. Afterward, the exact cross-linked positions need to be established. To this end, the cross-linked sample is typically enzymatically digested, followed by the identification of the resulting peptides.

**Figure 2:**
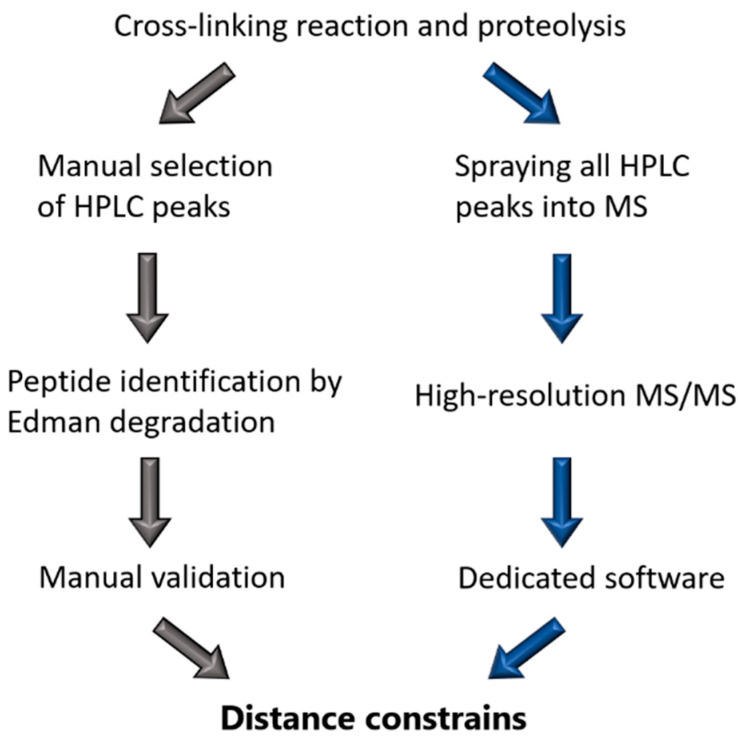
Comparison of cross-linking workflow of Steinert’s group (**left**) and the modern MS based method (**right**).

Back in the 1990s, Steinert’s group relied on N-terminal sequencing by Edman degradation to identify the cross-links [67] that have always been obtained through cross-linking with DST. Typically, they used the IF protein of interest in 10 mM triethanolamine-HCL pH 8.0 at 0.1 mg/mL and a ~100× molar excess of DST (Figure 2). Bands on the SDS-PAGE that corresponded to the cross-linked protein were subsequently cut out and digested with trypsin. Sometimes, multiple proteases were used to shorten the length of the peptides to facilitate their identification. Comparison of the elution profiles for control and cross-linked digests on reverse-phase HPLC was used as a primary means to identify the cross-linked products. These fractions were then treated with sodium periodate, which cleaves the glycol bond in the DST spacer group. Thereafter, the single peptides were rerun on HPLC to confirm a shift in retention time due to the cleaved interpeptide bond. Finally, the sequences of each peptide were derived using the Edman degradation reaction starting from the N-terminus of the peptide [10,50].

In contrast, the modern procedure to identify the cross-links is based on high-resolution MS. To this end, either the whole cross-linked sample or individual bands cut out of an SDS-PAGE gel are digested first with trypsin. The obtained peptide mixtures are then subjected to fractionation through liquid chromatography, typically followed by electrospray-ionization MS/MS [68] (Figure 2). Hereby, it is critical that high precision MS and fragment spectra are acquired. It should be noted that the use of a repertoire of cross-linkers including MS-cleavable ones as well as specific software tools typically enable a reliable assignment of a much larger number of cross-links than before, including those occurring at lower frequency [68,69,70].

### 3.3. Cross-Linking Based Models of Cytoplasmic IF Architecture by Steinert’s Group

Steinert and colleagues have reported chemical cross-linking of several cytoplasmic IF proteins including keratins K1/K10 and K5/K14, type III (vimentin and desmin), and type IV (α-internexin) proteins [10,50,56,71,72,73,74]. The cross-linking was performed in both low ionic strength conditions corresponding to soluble tetramers and after the addition of 150 mM KCl, which induced IF formation. Throughout the series of experiments, DST was used as the sole cross-linking agent, whereby only its main reactivity toward lysine residues was taken into account.

Most of the established cross-links involved residues of the coiled-coil rod domain at both ends. For instance, a total of 16 such cross-links were found for human vimentin [50]. Of note, some cross-links involving the head and tail domains were also detected. However, Steinert and colleagues argued that the cross-links within the rod were more valuable toward establishing the dimer–dimer alignments, since the rod structure could be fairly accurately predicted from the amino-acid sequence. Indeed, the coiled-coil segments could be approximated by a linear structure with 0.1485 nm per residue, which corresponds to a rise per residue in an α-helical coiled-coil [56,72]. In contrast, cross-links involving the flexible head and tail at least at one side were not used for modeling. Moreover, later papers of these authors did not even report such cross-links. Nevertheless, it should be noted that both head and tail domains are typically actively involved in the cross-linking reaction due the flexibility of both domains and high occupancy of target residues as evident from recent studies on lamins, for example (see below).

As a result, Steinert and colleagues have established that the overall cross-linking patterns were rather similar for type I/II (keratins), type III, and type IV filaments. Most importantly, the obtained cross-links could be consistently classified as belonging to one of three distinct modes of lateral alignment of dimers, A_11_, A_22_, and A_12_ (Figure 1B) [50,56,72,73]. The first mode could later be confidently assigned to the soluble tetrameric species as discussed already. For each of the three modes, the exact alignment of individual coiled-coil segments could be calculated as providing the best match to the experimental restraints. This alignment was then used to derive the lengths of the linkers connecting these segments.

**Figure 3:**
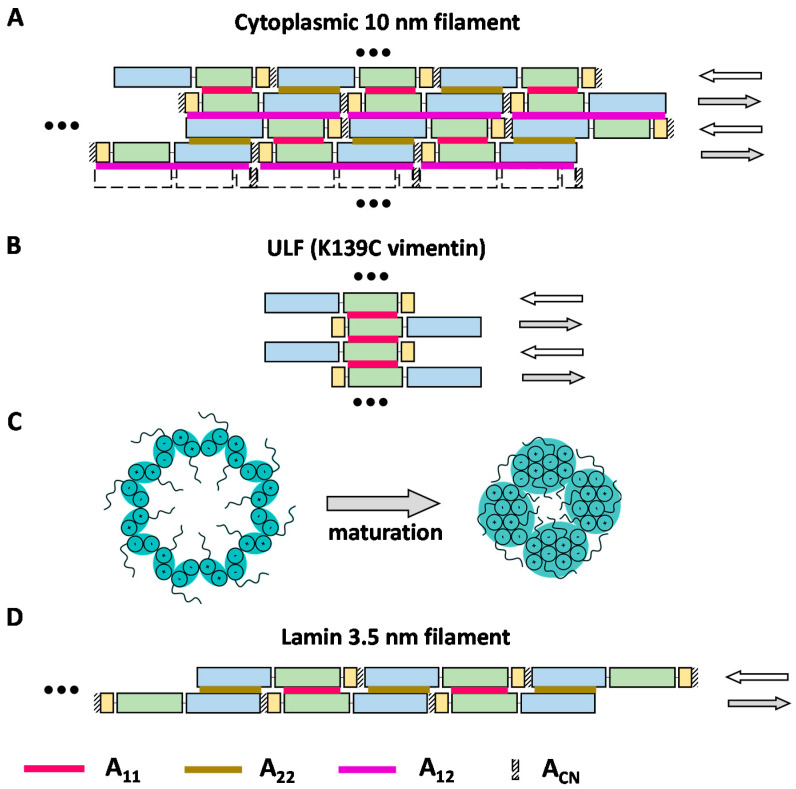
Proposed models of the IF structure. Coil1A, coil1B, and coil2 are schematically shown as yellow, green, and blue rectangles, respectively. Red, brown, and purple lines indicate A_11_, A_22_, and A_12_ contacts respectively. The A_CN_ contact between the C- and N-terminal ends of the rod domain is shown with diagonal stripes. (**A**) ‘Lattice-type’ model of dimer–dimer association within a cytoplasmic IF proposed by Steinert and colleagues. (**B**) Association of dimers into a ULF. (**C**) Possible maturation of the filament architecture viewed in the cross-section. The initial association of 16 dimers per cross-section eventually yields a more compact arrangement composed of four octameric protofilaments. Plus and minus signs denote the orientation of individual α-helices [77]. (**D**) Model of the lamin 3.5 nm filament containing two antiparallel threads of head-to-tail dimers.

Ultimately, the distance constraints were taken to propose a possible 2D arrangement of the dimers in the form of the so-called lattice model. To this end, the three lateral modes found were supplemented by the fourth mode A_CN_, which corresponds to a short ‘head-to-tail’ overlap of the C and N-ends of two parallel dimers that are longitudinally aligned (Figure 3A). It is the combination of the four modes that allowed the construction of the complete lattice. Of note, Steinert and colleagues never detected any cross-links corresponding to mode A_CN_ in cytoplasmic IF proteins. However, as may be seen from Figure 3A, this mode is a logical consequence of the coexistence of modes A_11_ and A_22_. Correspondingly, the overlap of the rod ends could be estimated at several nm for most proteins studied [72]. For instance, for vimentin, the axial periodicity was 42.7 nm according to the cross-linking data, while the rod domain length estimate used by Steinert and colleagues was 43.9 nm, yielding an A_CN_ overlap of ~1 nm [50].

Next, the 3D architecture of the filament was postulated to result from such lattice wrapping into a cylinder involving 16 dimers (Figure 3C). Indeed, scanning transmission EM on native and in vitro assembled vimentin filaments indicated that the filament cross-section typically contained 32 chains, even though a broader distribution including additional shoulder peaks has been observed [12,75]. 

The lattice model thus assumed that pairwise contacts of individual dimers mainly occurred on a 2D surface, as indeed such an arrangement could consistently explain the observed cross-links and the four dimer-dimer modes A_11_, A_22_, A_12_, and A_CN_. However, it should be stressed that even three decades later, there is still very limited 3D information that could confirm or reject the lattice model. Indeed, while some observations of vimentin and keratin IFs using (cryo)EM did suggest a hollow tube structure [76], direct visualization of individual dimers has thus far been unsuccessful. A later report of Steinert and colleagues [71] presented some additional cross-links that would go beyond the four canonical nearest-neighbor contacts in the lattice model. These additional cross-links seemed to support the possibility of further organization of the 2D lattice into octameric ‘protofibrils’ (Figure 3C), in line with earlier EM observations [76].

Cross-linking studies of the cytoplasmic ULFs were thus far limited to a single publication [10]. Here, the standard technique of Steinert was applied to the K139C mutant of vimentin, which was arrested at the ULF stage at 21 °C [10,20]. Interestingly, this study suggested that the ULFs predominantly contain A_11_ type contacts (Figure 3B). It should be noted that such arrangement does not allow a straightforward explanation. Indeed, for a simple longitudinal docking of such A_11_, only ULFs as rigid bodies cannot yield the complete palette of dimer–dimer contacts observed in mature filaments (A_11_/A_22_/A_12_/A_CN_), as may be seen from comparing Figure 3A,B. Instead, major rearrangements including sliding of individual dimers or tetramers would be necessary to enable the formation of all four contact types.

### 3.4. Cross-Linking Studies of Nuclear Lamins 

The last few years have seen a notable revival of cross-linking studies of IFs, now solely focused on lamins. Three recent papers [30,53,57] reported chemical cross-linking of in vitro assembled lamin A and its fragments as well as ex vivo lamin filaments [57]. These efforts are of great interest as lamin assembly is known to proceed quite differently compared to cytoplasmic IFs. As will be discussed here, the 3.5 nm lamin filaments [5] can in principle be described as a result of three types of dimer–dimer contacts, longitudinal A_CN_ and lateral A_11_ and A_22_ (Figure 3D). However, beyond the well-established A_11_ association, the limited results published to date for lamins do not yet provide a consistent and exhaustive snapshot of the relevant molecular interactions.

Historically, the characteristic tendency of lamin dimers to interact head-to-tail, resulting in longer dimeric threads in vitro, has been analyzed using glycerol spraying and rotary metal shadowing followed by EM [17]. Such threads for *Drosophila* lamin Dm_0_ revealed a periodicity of 52 nm [17]. Paracrystals could also be obtained, showing a periodicity of 24–25 nm, which seems to correspond to a half-staggered arrangement of dimers. At the same time, the theoretical length of the lamin rod domain is ~52 nm, as estimated from the total number of α-helical residues (350) and 0.1485 nm rise per residue in an α-helical coiled-coil. From these observations, the longitudinal (A_CN_) overlap in lamins could be calculated at ~2 nm. A study of complexes formed by N- and C-terminal human lamin A fragments [78] suggested a similar longitudinal overlap. However, more recent cryoelectron tomography (cryoET) studies of natively assembled lamin 3.5 nm filaments revealed a 40 nm periodicity in the tail domains, which hinted toward a much longer overlap of ~10 nm [5].

**Figure 4:**
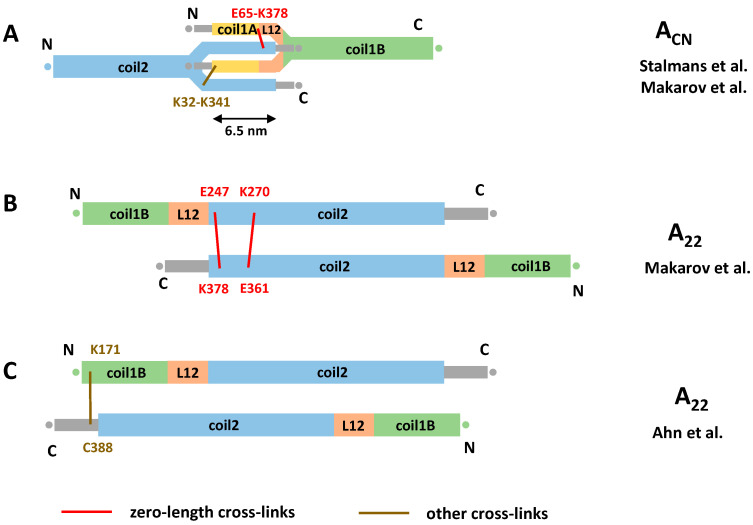
Dimer–dimer interactions for nuclear lamins as suggested from chemical cross-linking. (**A**) Longitudinal A_CN_ overlap as suggested by Stalmans et al. and Makarov et al. [30,57]. (**B**) Lateral A_22_ overlap as suggested by Makarov et al. (**C**) A_22_ overlap as proposed by Ahn et al. [53].

Recently, chemical cross-linking experiments were applied to explore the A_CN_ interaction of lamins. Compared to the work of Steinert, a wider repertoire of cross-linkers has been used including heterobifunctional ones. These studies have enabled a direct measurement of the A_CN_ overlap value for the first time. Two reports [30,57] consistently presented an overlap of ~6 nm (Figure 4A). It should be noted that this value is essentially based on the same single cross-link (E65-K378) obtained using EDC cross-linker, although additional cross-links involving the distal tail region were observed in the latter study. 

Interestingly, cross-linking results from Makarov et al. [57] for both in vitro assembled human lamin A filaments and ex vivo rat liver lamin filaments suggested that the total length of the lamin rod domain could be considerably shorter (41 nm) than generally thought (~52 nm as estimated above). It is this shortening that allows us to reconciliate the 40 nm periodicity observed by cryoEM [5] and the 6 nm overlap. More exactly, these authors found that a large fraction (~40%) of the detected cross-links could not be explained by the current dimer model. Instead, these cross-links would be satisfied by an assumption that three linkers (L1, L12, but also an extra linker in coil2) connected the coiled-coil segments in a ‘z’ fashion. This would reduce the total length of the rod domain. However, to date, no direct structural support for such mechanism is available. Indeed, crystallographic studies of lamin A fragments comprising linker L1 show that it is α-helical with limited flexibility [30,53]; likewise, a crystal structure including linker L12 [53] reveals this linker as α-helical, even though it is located close to the fragment end.

Despite the apparent lack of consensus on the length of the A_CN_ overlap in lamins, it could be speculated on the possible molecular mechanism of forming such an overlap. One possible explanation is unzipping of both coil1A and the C-terminal part of coil2 of the respective longitudinally interacting dimers and formation of an antiparallel four-helical assembly. The atomic model of the A_CN_ contact presented in [30] is compatible with this hypothesis (Figure 4A). In addition, the longitudinal A_CN_ assembly could be stabilized by the interaction between the flexible, positively charged head and tail domains and the acidic coiled-coil segments [17,79]. However, beyond some cross-links [57], little direct evidence toward such interaction is available to date.

Next, what concerns the lateral contacts between antiparallel lamin dimers, the EDC restraints obtained by Makarov et al. [57] have reliably pointed to the same A_11_ type interaction as revealed by X-ray crystallography, as discussed above. At the same time, the available data for the lateral A_22_ interaction are contradictory. On one hand, the data of Makarov et al. suggest a registered overlap of two antiparallel coil2 segments similar to the one seen for cytoplasmic IFs (Figure 3). Here, the N-terminus of the coil2 from one dimer is approximately aligned with the C-terminus of coil2 from another dimer (Figure 4B). On the other hand, chemical cross-linking of lamin A fragments by Ahn and co-workers [53] suggested a considerably longer overlap of the C-terminal halves of the rod. Here, the C-terminal portion (residues K171/K180/K181) of coil1B from one dimer was aligned with the start of the tail domain (residue R388) from the antiparallel dimer (Figure 4C). As the result of this supposedly longer A_22_ overlap and the ‘standard’ A_11_ overlap, Ahn et al. arrived at the A_CN_ overlap of as much as 14 nm. This value is principally compatible with both the 40 nm longitudinal periodicity of assembled 3.5 nm lamin filaments [5] and the standard value for the rod domain length (~51 nm), without the need to assume any ‘compression’ of the latter. 

## 4. Discussion and Outlook

As outlined here, studies of IF structure have resulted in considerable advances over the years, driven by a systematic application of several experimental methods such as X-ray crystallography and chemical cross-linking in particular. As a result, good progress has been achieved in establishing the three-dimensional structure of the elementary dimer of all IF types including both cytoplasmic and nuclear proteins. By now, crystallographic data have been obtained for a major part of the IF rod. In parallel, a confident understanding of the coiled-coil motif, in general, permits reliable 3D modeling also for the rod sections that could not be crystallized for any IF type. At the same time, only limited structural data could be obtained to date on the poorly ordered head and tail domains. 

Given these results, it may appear tempting to address the puzzle of the IF architecture by docking individual dimers/tetramers in silico in order to simulate the natural assembly process. While such attempts were made in the past, we are not aware of any sufficiently reliable predictions made along these lines. Indeed, IFs represent a highly complex and challenging object for structural studies. The main reason for that is a partial disorder seen at various levels. In particular, already, the elementary dimer is not entirely rigid. Beyond the intrinsically disordered N- and C-terminal regions, distinct structural flexibility of the rod domain has been proposed for nuclear lamins. Additionally, the assembled filaments of both nuclear and cytoplasmic proteins show a large degree of variability including varied number of subunits per cross-section and (most likely) a varied architecture. This has been observed for both in vitro assembled and native IFs [1,56,75,80].

Chemical cross-linking studies of cytoplasmic IFs by Steinert and colleagues were truly pioneering. Their lattice model hypothesis based on four distinct dimer–dimer association modes remains central to our current understanding of IF architecture. The attractiveness of this model is linked to its universality across cytoplasmic IF types, which appears logical, given the high conservation of the rod domain sequences. It should be noted that the A_11_ tetramer originally proposed from cross-linking data has been reliably confirmed by subsequent research as a major soluble species and thus an essential block of filament assembly. Studies of the A_11_ tetramers using X-ray crystallography, SDSL-EPR, and other methods have revealed a great number of atomic details that help to understand the subsequent assembly process. At the same time, two other types of lateral dimer–dimer interactions originally proposed by cross-linking studies (i.e., A_22_ and A_12_) remain far more elusive. In particular, attempts were made to draw conclusions from interdimeric contacts present in the crystal structures of coil2 fragments of keratin and lamin [81,82,83,84]. However, none of these hypotheses are sufficiently supported by cross-linking data or other independent evidence [72,83] and further studies are necessary. 

Hence, more research is still needed to substantiate the accuracy of the lattice model by applying orthogonal experimental methods. Moreover, the mechanism and pathway of the actual assembly process remains elusive. For instance, little is known on the changes in the dimer/tetramer structure that are likely to occur during assembly. Moreover, the mechanism of radial compaction that is the last assembly stage beyond longitudinal annealing of the ULFs is currently not understood at all. 

Moreover, the recent efforts to establish the molecular architecture of 3.5 nm lamin filaments have not yet produced a convincing picture. Here again, it was possible to reveal the A_11_ interaction in atomic detail. However, there is still a great deal of discrepancy across the recently published cross-linking studies with regard to both the A_22_ interaction and the longitudinal A_CN_ mode. This is somewhat surprising, given the fact that these filaments contain only two antiparallel threads of dimers and are therefore a much simpler system compared to 10 nm cytoplasmic IFs.

In our opinion, the vastly improved chemical cross-linking technique still holds great potential in the field. By combining the atomic structures of individual dimers or tetramers and inter-residual restraints provided by cross-linking, it should be possible to bootstrap in silico modeling of the complete filaments. The latter can be achieved using some recently proposed software such as the integrative modeling platform [85] and others. This approach is applicable not only to the WT filaments, but also to aberrant filaments, which are observed for many, although not all, disease-related mutants of various IF proteins [59,86,87,88,89,90]. One particular example is the L306R mutation in lamin A, which leads to “hyper-assembly” [91].

At the same time, it is clear that IFs present two major challenges that complicate such an approach. First, it would be highly beneficial to be able to cross-link distinct entities along the assembly pathway such as soluble cytoplasmic tetramers or mature assembled filaments (but also, ideally, the intermediates such as octamers and the ULFs [60]). However, the self-assembly of the filament is known to be readily triggered by relatively small changes in the environment, while the cross-linking procedure is not instantaneous and requires incubation in certain buffer conditions. Hence, the environmental conditions may interfere with the assembly process, making it challenging to reliably associate a set of cross-links with a distinct assembly stage. In a recent paper [57], cross-linking data corresponding to interdimer contacts have been obtained, even though the buffer system used to solubilize the protein stock was, in theory, only supporting soluble dimers. Furthermore, for cytoplasmic IFs, the cross-linking data suggested a gradual transition between several assembly states [50].

The second factor that particularly aggravates cross-linking studies is the conformational and stoichiometric disorder abundantly seen at various stages of IF assembly including the ULFs and mature filaments. As discussed above, multiple co-existing conformations are likely to result in numerous cross-linking events, especially if modern sensitive detection techniques are used [63,69]. In this scenario, the pool of the obtained cross-links cannot possibly be accounted for by a single rigid model, but complex multi-state molecular models should be considered. 

Importantly, we expected the obtained crystallographic data on IF fragments as well as cross-linking data on assembled filaments to be effectively enhanced through cryoelectron microscopy and cryoelectron tomography data. The advantage of these techniques is the ability to image the IF network or individual filaments in an unstained native-like state upon vitrification. While earlier cryoEM studies on several IF types could only provide limited insights [76], more recently, major technical improvements including pixel detectors, specimen preparation, and improved data analysis algorithms were introduced. This enabled major advances. For instance, in 2017, Turgay and co-workers presented cryoET on lamins expressed in vimentin-null mouse embryonic fibroblasts, revealing ~3.5 nm wide lamin filaments [5]. 

Still, as of today, cryoEM studies of IF networks are far from being trivial. Here also, their heterogeneity is a major obstacle. In a recent cryoEM study [61], in vivo assembled keratin networks were shown to feature significant diameter fluctuations even along a single filament. Moreover, the 10 nm cytoplasmic filaments appeared quite smooth and lack specific surface features. Here, picking the correct helical symmetry toward resolution enhancement through symmetry averaging is quite challenging, although the most recent results for vimentin filaments are indeed very encouraging [92]. As a result, cryoEM studies of IFs have not yet reached atomic resolution, which means that these data alone are insufficient to build reliable molecular models.

Despite these challenges, we strongly believe that the integrated use of (1) crystallographic data on the elementary dimer and A_11_ tetramer; (2) better cross-linking data that provide local inter-residual restraints; and finally cryoEM envelopes can bring distinct progress in resolving the IF structure enigma.
